# Sustainable deep learning-based breast lesion segmentation: impact of breast region segmentation on performance

**DOI:** 10.1186/s12880-025-01947-z

**Published:** 2025-10-10

**Authors:** Sam Narimani, Solveig Roth Hoff, Kathinka Dæhli Kurz, Kjell-Inge Gjesdal, Jürgen Geisler, Endre Grøvik

**Affiliations:** 1 Department of Physics, Norwegian University of Science and Technology, Trondheim, Norway; 2 Research and Development Department, More og Romsdal Hospital Trust, Aalesund, Norway; 3 Department of Radiology, More og Romsdal Hospital Trust, Aalesund, Norway; 4 Department of Health Sciences, Norwegian University of Science and Technology, Aalesund, Norway; 5 Stavanger Medical Imaging Group, Radiology Department, Stavanger University Hospital, Stavanger, Norway; 6 Department of Electrical Engineering and Computer Science, The University of Stavanger, Stavanger, Norway; 7 Department of Diagnostic Imaging, Akershus University Hospital, Lorenskog, Norway; 8 NordicCAD AS, Aalesund, Norway; 9 Institute of Clinical Medicine, University of Oslo, Lorenskog, Norway; 10 Department of Oncology, Akershus University Hospital, Lorenskog, Norway

**Keywords:** Breast region segmentation, Breast lesion segmentation, DCE-MRI, Deep learning

## Abstract

**Purpose:**

Segmentation of breast lesions in dynamic contrast-enhanced magnetic resonance imaging (DCE-MRI) is critical for effective diagnosis. This study investigates the impact of breast region segmentation (BRS) on the performance of deep learning-based breast lesion segmentation (BLS) in breast DCE-MRI.

**Methods:**

The study utilized the Stavanger Dataset, comprising 59 DCE-MRI scans, and employed the UNet++ architecture as the segmentation model. Four experimental approaches were designed to assess the influence of BRS on BLS: (1) Whole Volume (WV) without BRS, (2) WV with BRS, (3) BRS applied only to Selected Lesion-containing Slices (SLS), and (4) BRS applied to an Optimal Volume (OV). Data augmentation and oversampling techniques were implemented to address dataset limitations and enhance model generalizability. A systematic method was employed to determine OV sizes for patient’s DCE-MRI images ensuring full lesion inclusion. Model training and validation were conducted using a hybrid loss function—comprising Dice loss, focal loss, and cross-entropy loss—and a five-fold cross-validation strategy. Final evaluations were performed on a randomly split test dataset for each of the four approaches.

**Results:**

The findings indicate that applying BRS significantly enhances model performance. The most notable improvement was observed in the fourth approach, BRS with OV, which achieved approximately a 50% increase in segmentation accuracy compared to the non-BRS baseline. Furthermore, the BRS with OV approach resulted in a substantial reduction in computational energy consumption—up to 450%, highlighting its potential as an environmentally sustainable solution for large-scale applications.

## Introduction

Breast cancer is the most common cancer among women and the second leading cause of cancer-related death in women worldwide [1]. Although the number of deaths due to breast cancer has slightly decreased over the past 30 years in Norway, 619 women still lost their lives to the disease in 2022 [2, 3]. Therefore, continuous research into new methods for improving the detection and characterization of breast cancer is essential to further reduce mortality.

Medical imaging plays a crucial role in the detection and diagnosis of breast cancer. Commonly used imaging modalities include mammography, ultrasound, and magnetic resonance imaging (MRI). Among these, dynamic contrast-enhanced MRI (DCE-MRI) has demonstrated the highest sensitivity. It is primarily employed for staging confirmed malignancies, evaluating response to neoadjuvant chemotherapy, and screening individuals at elevated risk [4, 5]. Meanwhile, Artificial Intelligence (AI) has shown increasing potential in breast cancer detection and diagnosis [6, 7]. AI algorithms can analyze breast DCE-MRI and effectively detect, segment, and classify abnormalities in breast anatomy [8–10]. Among all AI tasks, segmentation plays a critical role in breast cancer detection and characterization, as it helps localize lesions and reduce ambiguity by isolating regions of interest (ROI), thereby enabling quantitative analysis [11, 12]. Numerous studies on breast cancer segmentation have utilized machine learning (ML) and deep learning (DL) models [13–15]. Notably, Convolutional Neural Networks (CNNs) [16–19], transformers [20, 20], and ensemble models [21] have been widely adopted in this area of research. Among DL models, CNN-based architectures have garnered significant attention. One of the most well-known models, UNet [22], was introduced in 2015 and has paved the way for advanced variants such as Connected-UNet [23] and UNet++ [24]. Furthermore, transformer-based UNet variations have demonstrated superior performance [20]. Despite these advancements, breast lesion segmentation (BLS) still faces several challenges, such as class imbalance and intensity similarity with breast tissue [25].

Many DL models have been validated for BLS using DCE-MRI, but few explore how breast region segmentation (BRS) affects BLS. Additionally, existing DL models often come with high computational costs, which contribute negatively to environmental sustainability. Therefore, this study aims to investigate whether BRS can enhance BLS performance and reduce both training time (TT) and carbon footprint (CFP).

## Literature and methodology

### Literature

Breast cancer segmentation in medical imaging, especially in DCE-MRI, has received growing attention over the past decade. Researchers have explored various approaches to improve segmentation efficiency. These include advanced preprocessing techniques, DL models such as U-Net variants and transformers, and different optimization strategies. Table 1 provides a summary of recent studies in the field, highlighting the innovative methodologies employed and the evaluation metrics used.

**Table 1:** Summary of latest research in BLS

Authors (Year)	Methods	Evaluation Metrics
Zhang et al. [16] (2018)	FCN-UNet for BRS and BLS	Dice, Sensitivity
Jiao et al. [17] (2020)	UNet++ for BRS and Faster RCNN for lesion detection	Dice, Jaccard
Qin et al. [26] (2022)	Joint Transformer and UNet for BRS and BLS	Dice, Jaccard
Pandey et al. [27] (2022)	Denoising and and filtering methods with agraph-based approach	Dice, Jaccard
Qin et al. [20] (2022)	Refined UNet	Dice, Jaccard
Khaled et al. [21] (2022)	Ensemble-based UNet	Dice
Igbal et al. [28] (2023)	Swin Transformer for breast tumor segmentation	Dice
Huang et al. [18] (2023)	SegNet for BRS and BLS	Dice, Jaccard
Zhang et al. [29] (2023)	Semantic-aware transformer for BLS	Dice, Jaccard
Zhou et al. [30] (2024)	Hybrid DL Networks with two-stage optimization	Dice, Sensitivity
Babu et al. [31] (2025)	Contextual Regularization-Based Energy Optimization model	Dice, Jaccard
Star et al. [19] (2025)	DeepLabV3 with modified ResNet50	Dice, Jaccard
Zhong et al. [32] (2025)	Weakly-supervised BLS	Dice, Jaccard
Wang et al. [33] (2025)	Transformer-based explainable model for BLS	Dice, Sensitivity

### Data pre-processing and insight

#### Data pre-processing

**Fig. 1:**
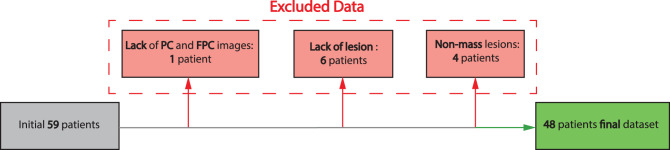
Process of data exclusion based on available information in Stavanger cohort (red and green lines indicate exclusion and inclusion of data, respectively)

The dataset utilized in this study, referred to as the Stavanger Dataset, was obtained from Stavanger University Hospital. A comprehensive description of its characteristics and imaging features is available in our previous work [34]. The dataset comprises sequences from breast DCE-MRI, specifically including Pre-Contrast (PC) and First Post-Contrast (FPC) images. Initially, the dataset included scans from 59 patients; however, 11 cases were excluded due to missing or incomplete data. Consequently, the final study cohort consisted of 48 patients with complete and relevant imaging data for further analysis. The stepwise exclusion criteria are illustrated in Fig. 1.

To standardize the data preparation pipeline, the PC and FPC images were automatically identified for each patient. These images were then converted from the Digital Imaging and Communications in Medicine (DICOM) format—widely used in medical imaging—to the Neuroimaging Informatics Technology Initiative (NIFTI) format, which is compatible with the DL models used in this study. The pipeline also included a mechanism to detect missing data, such as the absence of either PC or FPC images. Random oversampling was applied to ensure consistent volumetric representation across the dataset. This not only enhanced data uniformity but also simplified the model’s data-loading process. Additionally, all images were reoriented to the standard Right-Anterior-Superior (RAS) coordinate system, which is commonly adopted in breast imaging within Scandinavian clinical practice.

**Fig. 2:**
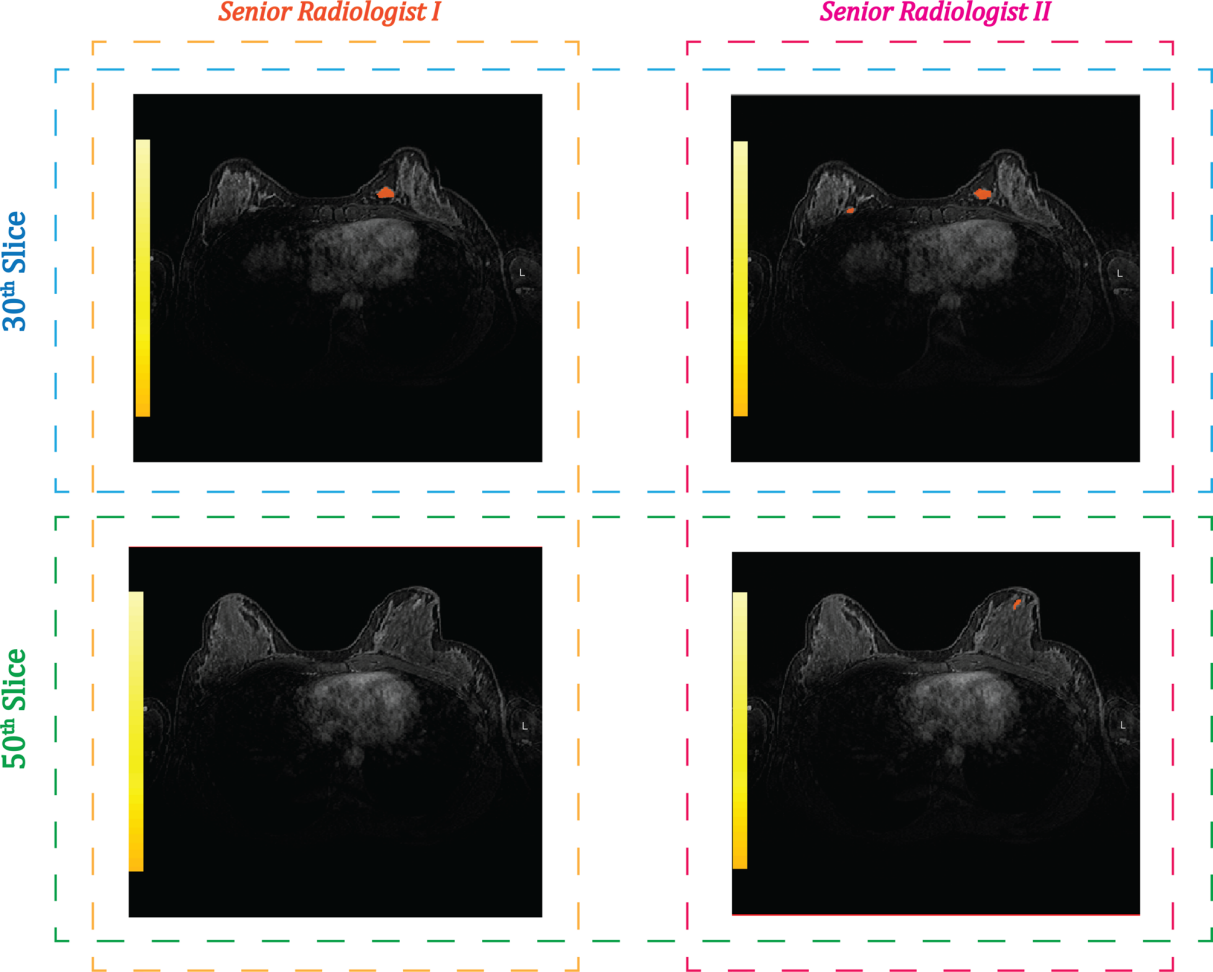
Annotation comparison between two senior radiologists on a patient in test dataset

Additionally, subtraction images, along with the input data—PC and FPC—were incorporated into the data processing pipeline as needed. Lesion annotations were performed by an experienced senior breast radiologist with extensive expertise in breast cancer diagnostics. For the training datasets, only the largest lesion per patient was annotated to focus model learning on the most clinically significant region. In contrast, the test datasets were annotated by a second senior breast radiologist to ensure that all detectable lesions were included, thereby enabling a comprehensive evaluation of the model’s performance. Figure 2 illustrates the annotation example for a patient in the test dataset, performed by two senior breast radiologists. As depicted, Radiologist I annotated only the largest lesion and was therefore excluded from the test evaluation. In contrast, Radiologist II annotated all detectable lesions, ensuring a comprehensive and unbiased evaluation of the test dataset.

#### Data insights

**Fig. 3:**
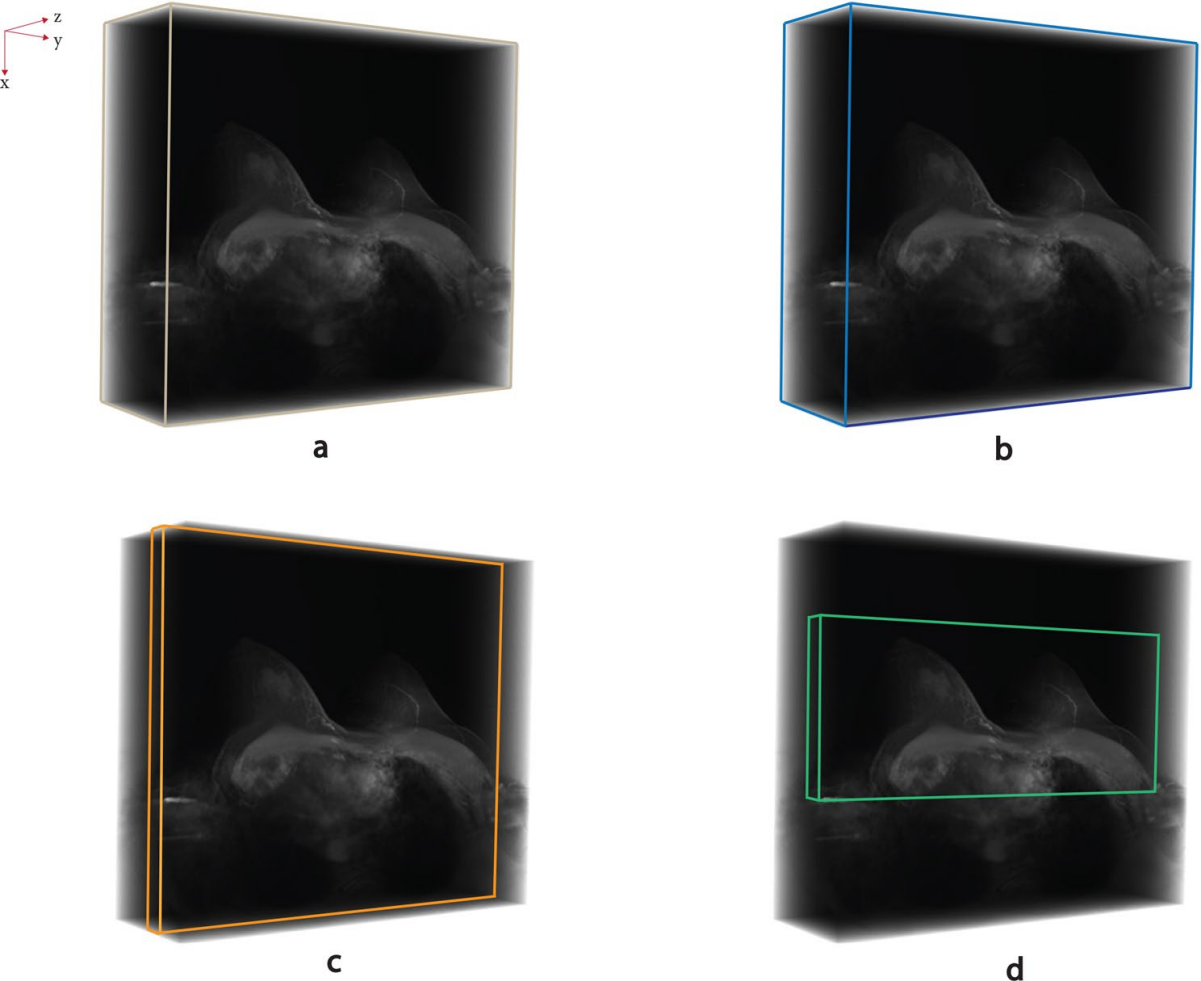
Visual depiction of the image regions employed by each method: (**a**) and (**b**) represent WV, whereas (**c**) and (**d**) correspond to SLS and OV, respectively

This study primarily concentrates on effect of BRS on BLS. To investigate this, two separate groups of BLS approaches were considered: one with BRS applied to all images and the other without BRS. However, this problem could be explored further using a more sustainable and environmentally friendly approach by incorporating additional data analysis steps. Therefore, after applying BRS to both PC and FPC images, the study explores three potential volume strategies: the Whole Volume (WV), the Selected Lesion-containing Slices (SLS)—which include only the slices containing lesions—and The Optimal Volume (OV), which incorporates 2D slice optimization in SLS. Consequently, four different datasets including original data with WV, BRS with WV, BRS with SLS, and BRS with OV were created for this study. These four types of datasets, utilized separately in DL models, are illustrated in Fig. 3. Notably, the shape of the label files corresponds directly to the structure of their respective input datasets.

#### Slice optimization

To facilitate a more detailed examination of the methodology involving BRS, a previously developed pretrained model was employed for whole breast segmentation using BRS. The segmentation masks generated by BRS were applied to the corresponding images, resulting in a new set of images wherein extraneous noise from low-intensity anterior regions was excluded, and posterior organs such as the heart and lungs were removed. This isolation of breast anatomy enables a more precise analysis of the internal breast structure. By restricting the focus exclusively to the breast tissue, it becomes feasible to optimize the localization of potential lesion sites.

To determine the optimal height for analysis, a detailed data evaluation was conducted across all images to ensure the inclusion of all lesions within the designated region. For each patient, the first non-zero pixel was identified from both the superior and inferior bounds of the image slices. Subsequently, the maximum and minimum spatial coordinates were calculated across all slices for each patient. In addition, the maximum depth of the breast along the body midline was assessed to establish a cropping boundary that maintains a safe margin, thereby ensuring that all lesions remain within the cropped region.

**Fig. 4:**
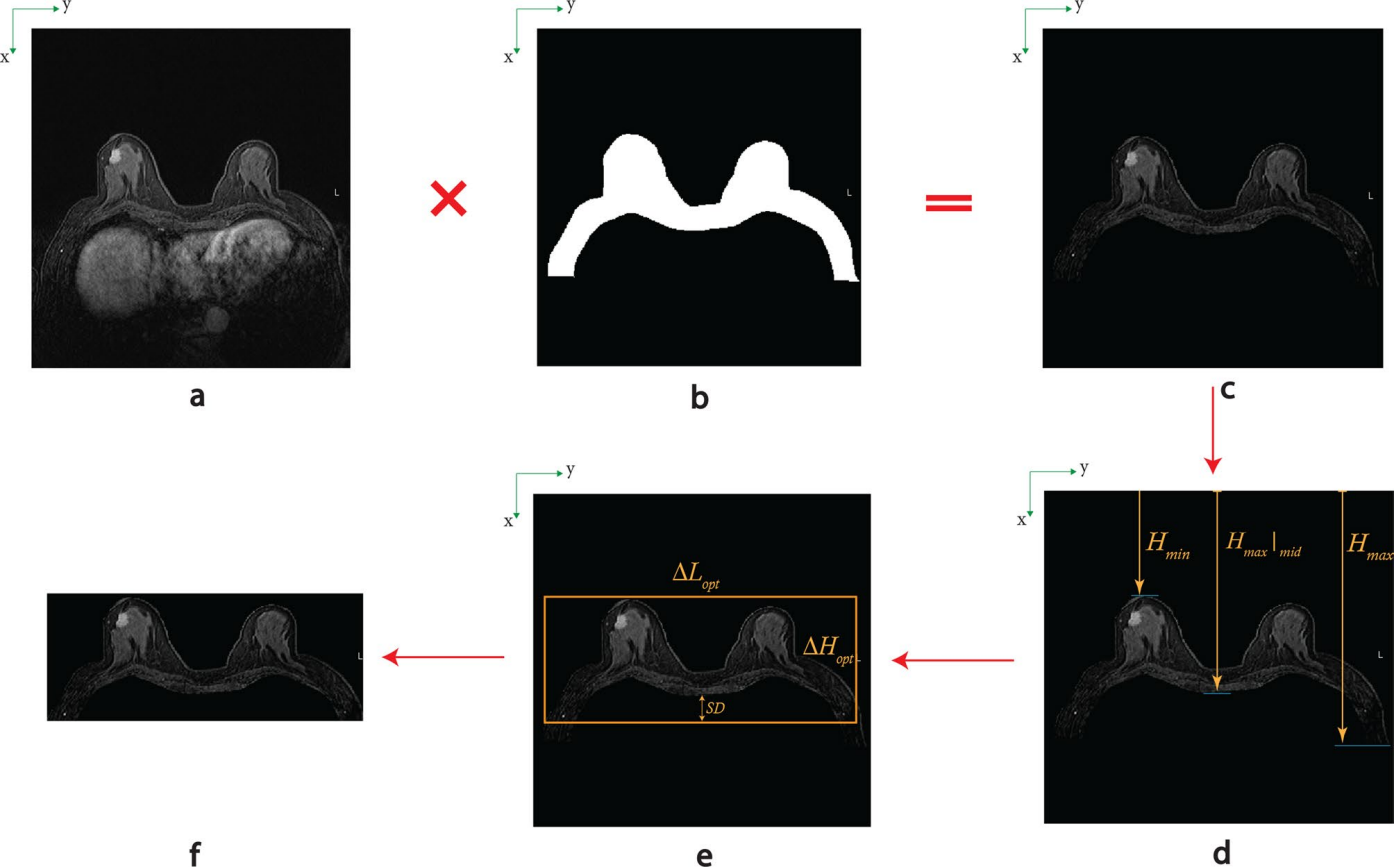
Process of image size optimization (**a**: original PC and FPC images, **b**: mask file of corresponding patient predicted by pre-trained BRS model, **c**: new images by multiplying original and corresponding predicted mask file, **d**: finding the maximum and minimum of non-zero pixels in image and middle of breast chest, **e**: optimizing the rectangle containing the ROI (SD stands for safe distance), **f**: final slice for training of OV)

Based on these measurements, the maximal height encompassing the ROI across all patients was selected and applied uniformly. Furthermore, adjustments were made to the input dimensions of the DL model to be multiples of 32, conforming to architectural requirements of the current DL framework and ensuring compatibility with widely utilized segmentation networks for prospective applications. Figure 4 illustrates the workflow for refining the images to produce a more focused and compact ROI, thereby enhancing the efficiency and accuracy of subsequent analyses.

#### Overlay map

To construct an overlay map from a collection of mask files within a dataset, each individual mask must be aggregated through pixel-wise summation. This results in a composite representation that reflects the cumulative spatial distribution of all annotated regions across the dataset. The resulting overlay map can then be visualized using an appropriate colormap, facilitating the identification of areas with high annotation density. Such visualizations are instrumental in revealing patterns of label distribution, which can provide insights into anatomical structures and spatial labeling tendencies. This methodology is particularly valuable in medical imaging, where understanding the frequency and location of annotated regions can support both retrospective anatomical analysis and the development of robust DL models for clinical applications. The overlay map intensity is mathematically defined in Eq. 1, where $M_{p,s}(x, y)$ denotes the binary mask value at pixel location $(x, y)$ for image slice $s$ in patient $p$, across $m$ patients and $n$ image slices per patient.

1 $$\mathcal{I}_{\text{Overlay}}(x, y) = \sum_{p=1}^{m} \sum_{s=1}^{n} M_{p,s}(x, y)$$

**Fig. 5:**
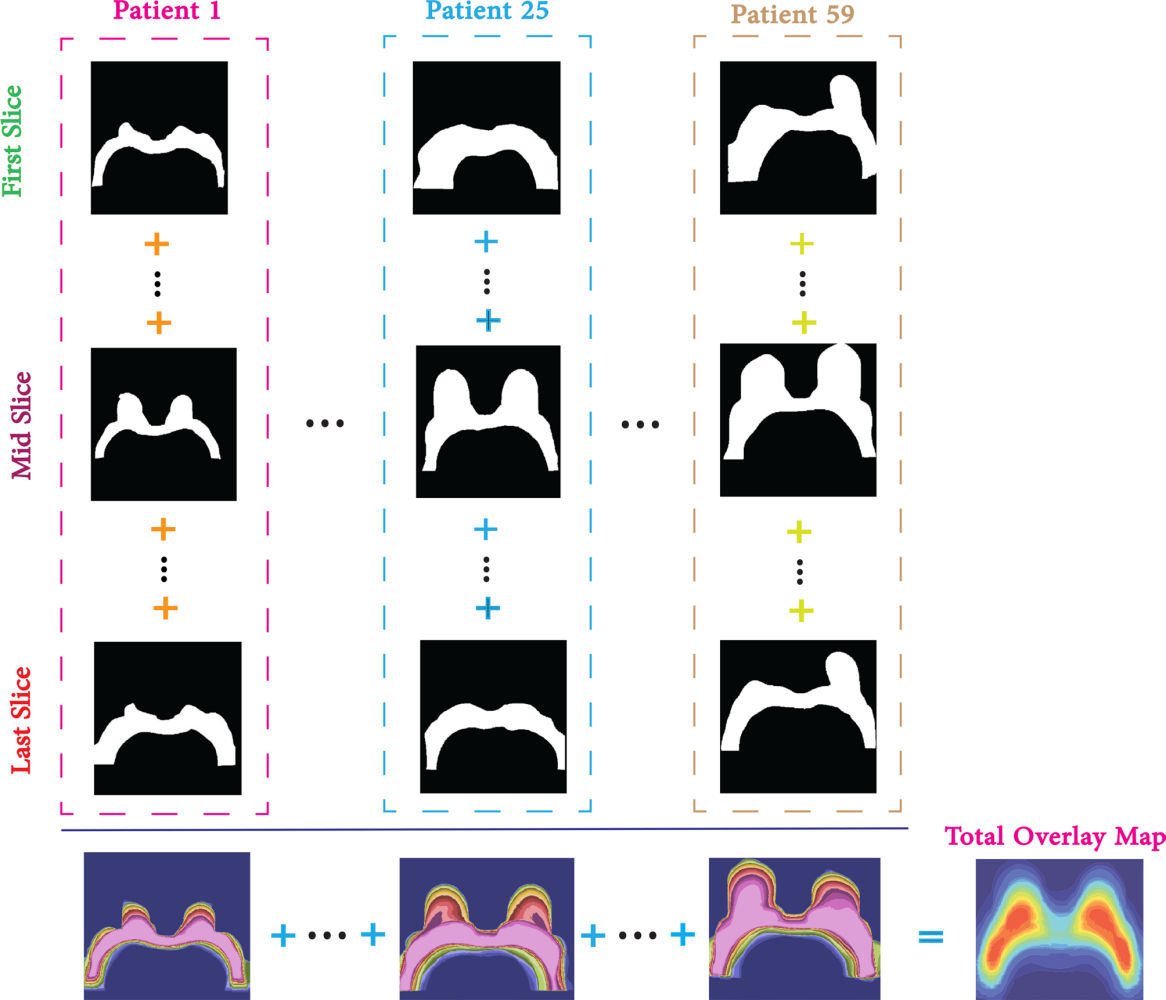
Overlay map process for whole breast labels

Figure 5 provides a detailed illustration of the overlay map construction process, demonstrating how individual mask contributions are combined to produce a detailed visualization of annotation density for the whole dataset.

### DL network

DL segmentation models such as UNet and UNet++ are widely used for segmenting small objects, including lesions, primarily relying on CNNs [35–37]. These models follow a contraction-expansion process, commonly known as an encoder-decoder architecture. The encoder is responsible for extracting and identifying the most relevant features at each encoding level, while the decoder reconstructs these compressed features to produce the desired output. Skip connections between the encoder and decoder help retain spatial information lost during downsampling, thereby enhancing model performance and segmentation accuracy [22, 24].

**Fig. 6:**
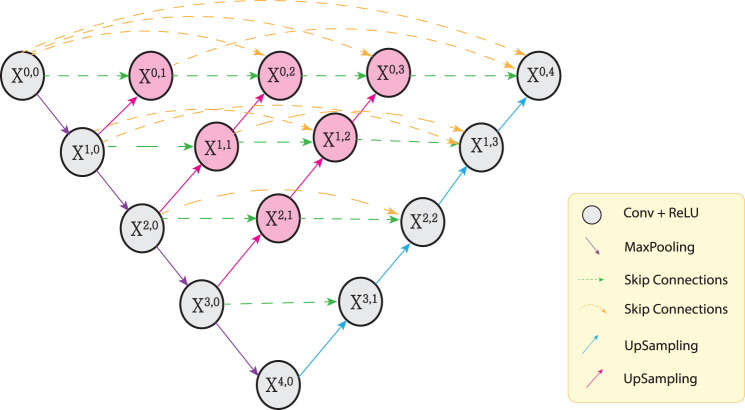
Diagram of UNet++ architecture showcasing its nested and densely connected skip pathways

Among encoder-decoder architectures with skip connections, UNet++ stands out for its nested design, which improves input-output correspondence [38, 39]. These nested skip connections help preserve critical features during transmission between the encoder and decoder, leading to enhanced overall performance and more accurate segmentation results [24]. The overall structure of the UNet++ architecture, including its nested and densely connected skip pathways, is depicted in Fig. 6. Further details—such as the number of learnable parameters, network depth, layer types, and other distinctive features—are summarized in Table 2.

**Table 2:** Unet++ model specifications

Category	Feature	Details
	Number of Parameters	2410468
General Information	Input Shape	(H, W, C)
	Output Shape	(H, W, 1)
	Depth of Network	5 levels
Network Architecture	Number of Layers	240
	Base Filter Size	32
	Convolution Type	3x3 Convolutions with stride 1
Layer Types	Pooling Layers	MaxPooling 2x2
	Upsampling Layers	Transposed Convolution
	Activation Function	ReLU for hidden layers, Sigmoid for output
Activation and Regularization	Normalization	Batch Normalization
	Dropout	0.00

### Evaluation and setup

To evaluate the performance of the DL model during training, a hybrid loss function was employed in conjunction with 5-fold cross-validation. The hybrid loss function used in the training process is presented in Eq. 2.

2 $$\begin{aligned}\mathcal{L}_{\text{Hybrid}} &= \alpha \cdot \mathcal{L}_{\text{Dice}} + \beta \cdot \mathcal{L}_{\text{Focal}} + \gamma \cdot \mathcal{L}_{\text{Cross-Entropy}} \end{aligned}$$

The coefficients $\alpha$, $\beta$, and $\gamma$ represent the contributions of Dice loss, Focal loss, and Cross-Entropy loss, respectively, with the constraint $\alpha + \beta + \gamma = 1$. These parameters are empirically tuned according to task requirements. For instance, increasing the Focal loss coefficient mitigates class imbalance, while emphasizing Dice loss enhances segmentation quality. In this study, the coefficients were set as $\alpha = 0.1$, $\beta = 0.45$, and $\gamma = 0.45$. The definitions of Dice loss [40], Focal loss [41], and Cross-Entropy loss [42] are provided in Eqs. 3, 4, and 5, respectively.

3 $$\mathcal{L}_{\text{Dice}}(P, G) = 1 - 2 \cdot \frac{|P \cap G|}{|P| + |G|}$$

4 $$\mathcal{L}_{\text{Focal}}(p_t) = - \sum \alpha_t \cdot (1 - p_t)^{\gamma_f} \cdot \log(p_t)$$

5 $$\mathcal{L}_{\text{Cross-Entropy}}(P, G) = - \sum G \cdot \log(P)$$

Dice loss measures the overlap between the predicted segmentation $P$ and the ground truth $G$ [40]. Focal loss addresses class imbalance by down-weighting well-classified examples. In Eq. 4, $p_t$ denotes the predicted probability for the true class, $\alpha_t$ is a balancing factor, and $\gamma_f$ is a focusing parameter that emphasizes harder-to-classify samples [41]. Cross-Entropy loss, commonly used in classification tasks, calculates the divergence between the predicted distribution $P$ and the true distribution $G$. It penalizes incorrect predictions more heavily to align predictions with the ground truth [42].

To assess the results on the test dataset, different metrics such as Dice, IoU, Precision, and Recall are used. These metrics are defined in Eqs. 6 to 9.

6 $$\text{Dice} = \frac{2TP}{2TP + FN + FP}$$

7 $$\text{IoU} = \frac{TP}{TP + FN + FP}$$

8 $$\text{Precision} = \frac{TP}{TP + FP}$$

9 $$\text{Recall} = \frac{TP}{TP + FN}$$

Where $TP$, $FP$, and $FN$ denote True Positives, False Positives, and False Negatives, respectively.

Additionally, the carbon emission is an important consideration in DL applications [43]. The production of 1 kWh of energy results in an average CFP of 475 gCO$_2$ [44]. Therefore, the CFP for each fold can be calculated using Eq. 10:

10 $$\text{CFP} = \frac{0.475 \cdot \text{TT}}{3600}$$

Where $\text{CFP}$ and $\text{TT}$ are in kilograms of CO$_2$ for each fold and seconds, respectively. To better visualize the impact of CFP, the normalized CFP is defined as shown in Eq. 11:

11 $$\text{Norm}_{\text{CFP}} = 1 - (\text{CFP}_{\max} - \text{CFP}_{\min}) \left(\frac{\text{CFP}}{\text{CFP}_{\max}}\right)$$

Where $\text{Norm}_{\text{CFP}}$ indicates that a higher value corresponds to a more efficient approach.

The experimental setup used in this study is identical to that of the previous work [34], with the exception of an upgrade to 64 GB of RAM. As a result, the total energy consumption remains approximately the same, estimated at 1 kWh per DL training session.

**Fig. 7:**
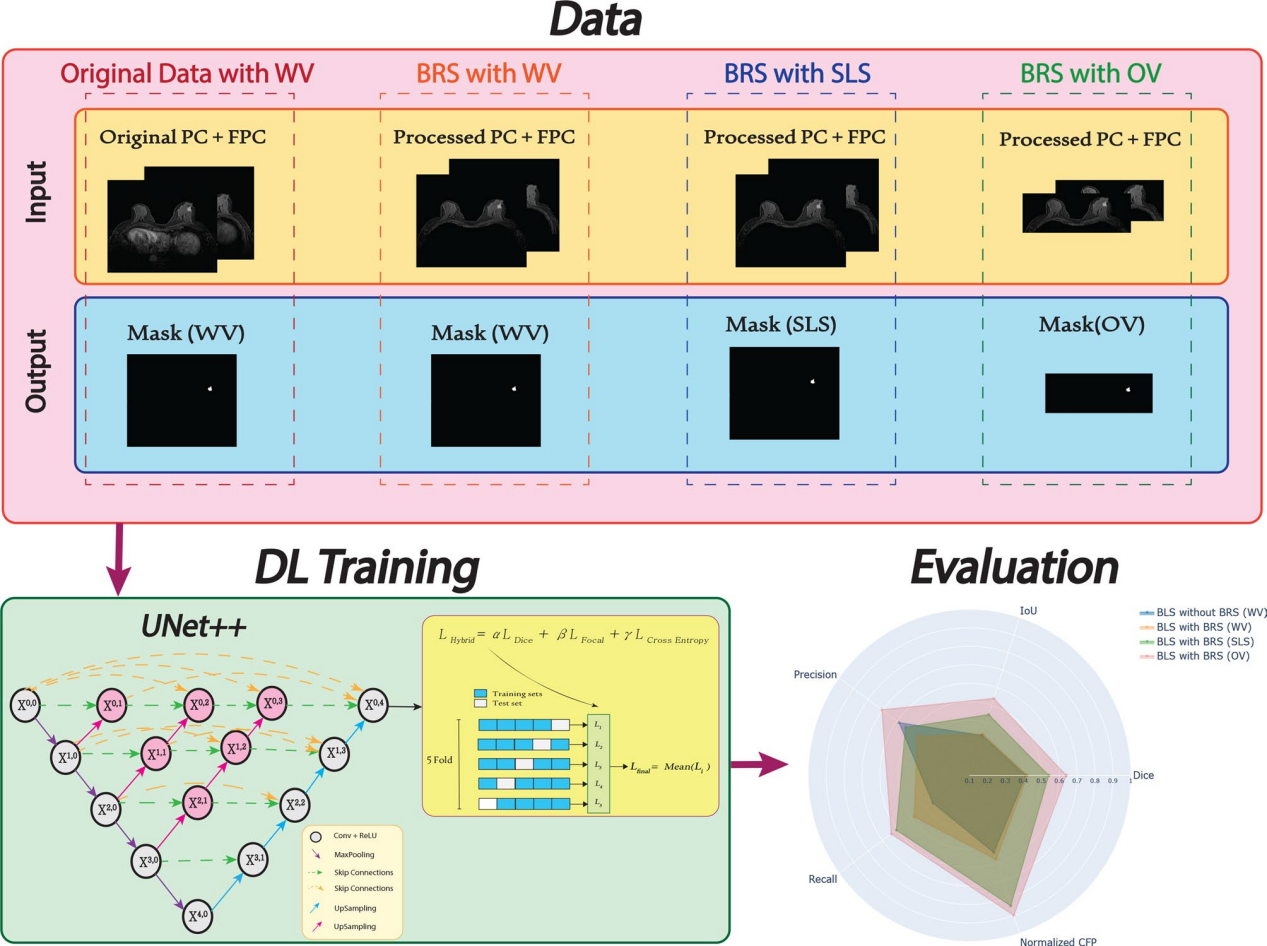
Overview of data configurations (WV without BRS, WV with BRS, SLS with BRS, and OV with BRS), the UNet++ model, a hybrid loss function (dice, focal, and Cross-entropy), and evaluation metrics (IoU, dice, precision, recall, and normalized CFP)

Figure 7 provides an overview of the data types, DL model, the use of 5-fold cross-validation with the hybrid loss function and the evaluation metrics described above.

## Results

### Experiments

The input data consisted of PC and FPC images, along with their corresponding masks, which served as the baseline outputs. As outlined in the data insights subsection, both the input images and their respective masks vary in shape depending on the corresponding approach. Table 3 summarizes the final NIFTI dimensions for each patient across all approaches used during training. As shown, the BRS with OV approach contains approximately 70% fewer slices compared to methods that utilize the full volume.

**Table 3:** Data shape across individual patients

Approach	Original Data with WV	BRS with WV	BRS with SLS	BRS with OV
Input Shape	(352,352,150)	(352,352,150)	(352,352,**42**)	(352,**192**,**42**)

The training process involved the 2D UNet++ model using 5-fold cross-validation combined with the hybrid loss function. Training was conducted on a slice-by-slice basis, where each slice was independently fed into the model, reflecting the use of a two-dimensional framework. To minimize the loss during each epoch, the RAdam optimizer was employed with an initial learning rate of 0.001, in combination with a ReduceLROnPlateau scheduler. This scheduler adaptively adjusted the learning rate based on validation performance, aiming to boost convergency and performance of model. Across all approaches, a batch size of 8 was used, and data shuffling was applied only during training. To evaluate model performance on previously unseen data, a random subset of two patients was held out as the test set. All other hyperparameters remained constant across experiments to enable a precise evaluation of the impact of the studied variables.

### BRS effect and slice optimization

**Fig. 8:**
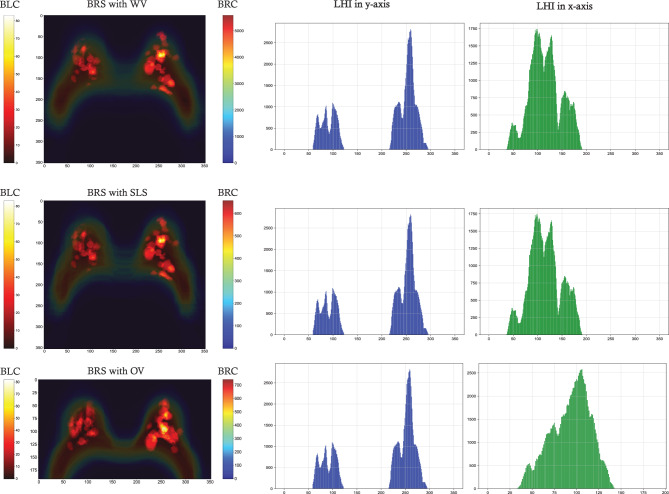
Overlay maps showing the aggregated lesion masks superimposed on the corresponding breast region masks (BLC, BRC, and LHI refer to Breast Lesion Colorbar, Breast Region Colorbar, and Lesion Histogram Intensity, respectively.)

To assess the impact of BRS and subsequent data analysis on the segmentation outcomes, we examine the final three datasets depicted in Fig. 3, all of which incorporate BRS-predicted masks. By overlaying lesion labels on the corresponding whole breast masks, we can better visualize how both breast regions and lesions are distributed across the image slices. These overlay maps are displayed in Fig. 8, arranged vertically by method. Complementing these visualizations, lesion distribution histograms are provided along both the x- and y-axes for each approach. The overlay maps corresponding to BRS with WV and BRS with SLS demonstrate comparable lesion distributions, reflecting the similarity of their underlying lesion masks. However, the associated breast region masks exhibit marked differences, as evidenced by the varying ranges in their color bars. Notably, the BRS with SLS map includes fewer slices near the body midline, indicating a more limited spatial coverage compared to the BRS with WV approach. In contrast, the BRS with OV overlay map exhibits a more compact and anatomically coherent distribution of both the breast region and lesion masks. Specifically, the breast region appears more spatially constrained, while the lesions are more precisely localized within each breast. These findings underscore the distinct characteristics of each segmentation method, with the BRS with OV approach showing potential advantages in producing anatomically consistent and spatially focused segmentations.

The lesion histogram along the y-axis reveals that lesions are predominantly concentrated in the left breast. In the x-axis direction, the BRS with OV approach demonstrates a more compact lesion distribution compared to the BRS with WV and BRS with SLS approaches. Specifically, lesions in the BRS with WV and SLS methods are centered around $x = 190$, whereas in the BRS with OV approach, they shift to approximately $x = 140$. This shift indicates a reduced SD in lesion distribution in the BRS with OV method relative to the others.

**Fig. 9:**
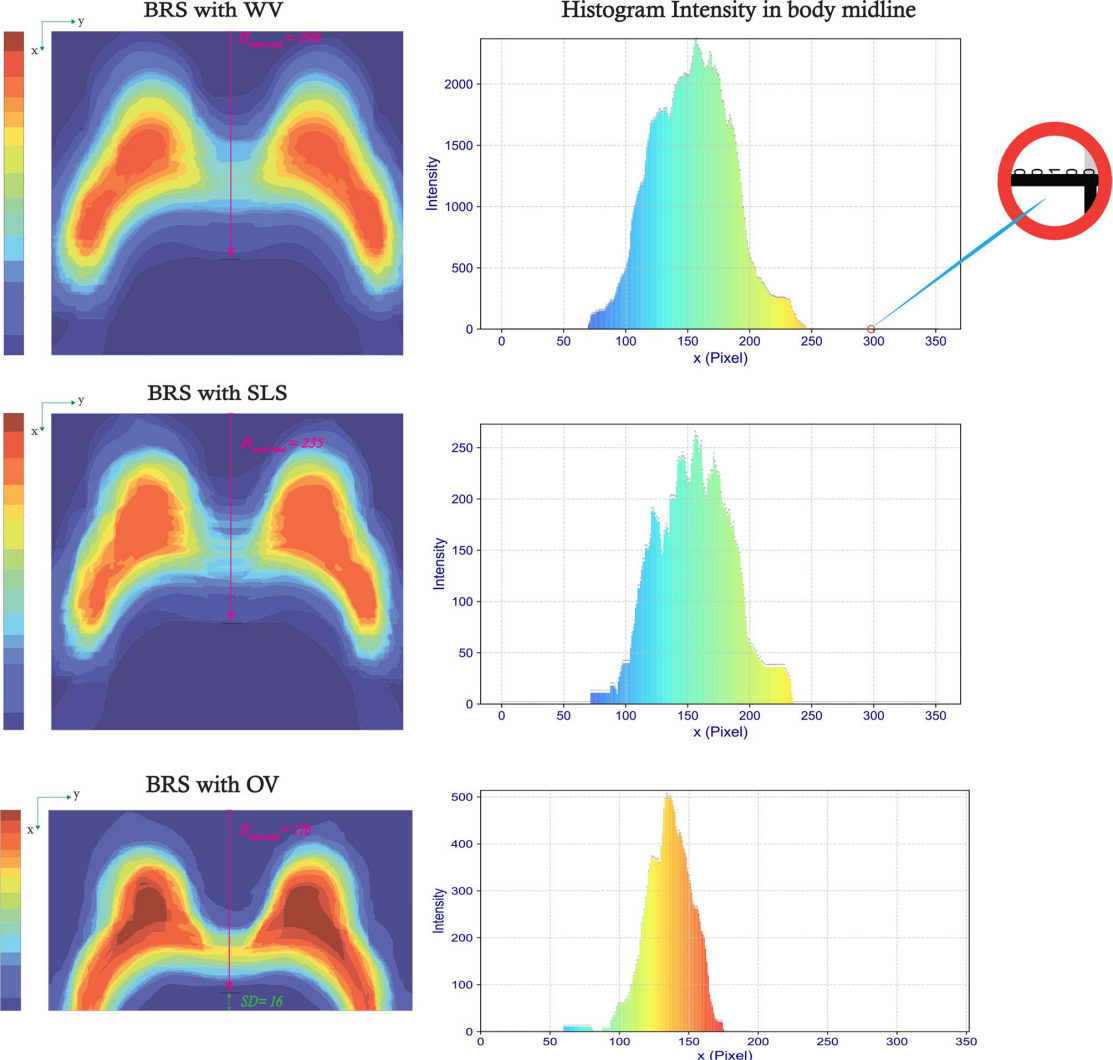
The left column displays overlay maps of the breast region for all patients, corresponding to each respective method. The right column presents intensity histograms along the body midline, parallel to the x-axis. The highlighted (zoomed-in) region indicates a single pixel identified during analysis, with a maximum midline intensity value of $H_{\text{max} \vert \text{mid}} = 298$

Furthermore, it is important to assess the overlay map of breast region along the body midline, particularly in the direction parallel to the x-axis. This analysis can reveal a broader range for $H_{\text{max} \vert \text{mid}}$, the maximum breast region presence at the midline, compared to the overlay map for lesion labels. As illustrated in Fig. 9, the BRS with WV approach exhibits the highest $H_{\text{max} \vert \text{mid}}$, with a value of 298, followed by the BRS with SLS method at 235. In contrast, the BRS with OV approach exhibits the lowest value, reaching only 176, thereby indicating the most limited vertical extent among all evaluated methods.

Finally, based on the model configuration requirements and the observed distribution in the BRS with OV method, a midline size of 192 pixels was selected. This corresponds to a SD of 16 pixels—approximately 1.6 cm—from the center of the chest along the x-axis. This selection provides sufficient coverage of the breast region while preserving a spatial range that is anatomically consistent and constrained.

### Model performance and generalization

Model performance and generalizability for each approach are summarized in Table 4, based on training and validation loss. The first approach, BLS without BRS, demonstrated the weakest performance, with training and validation losses of $0.0962 \pm 0.0012$ and $0.0945 \pm 0.0020$, respectively. Similarly, BLS with BRS (WV) yielded comparably poor results, with losses of $0.0948 \pm 0.0003$ for training and $0.0924 \pm 0.0014$ for validation. In contrast, the BLS with OV approach achieved the most favorable outcomes, attaining significantly lower losses of $0.0339 \pm 0.0062$ and $0.0389 \pm 0.0057$ in training and validation, respectively. The third approach, BLS with SLS, produced intermediate performance, with training and validation losses of $0.0462 \pm 0.0041$ and $0.0442 \pm 0.0034$, respectively.

**Table 4:** Training and validation hybrid loss for different approaches evaluated using 5-fold cross-validation

Approach	Training Loss	Validation Loss
BLS without BRS (WV)	0.0962 $\pm$ 0.0012	0.0945 $\pm$ 0.0020
BLS with BRS (WV)	0.0948 $\pm$ 0.0003	0.0924 $\pm$ 0.0014
BLS with BRS (SLS)	0.0462 $\pm$ 0.0041	0.0442 $\pm$ 0.0034
BLS with BRS (OV)	0.0339 $\pm$ 0.0062	0.0389 $\pm$ 0.0057

**Fig. 10:**
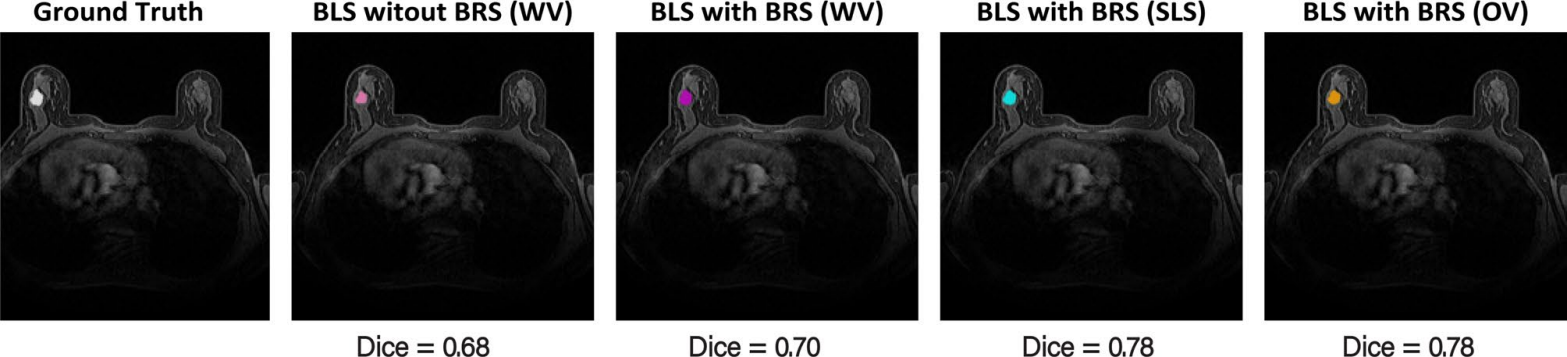
Comparative prediction performance of various methods on the test dataset

Figure 10 depicts prediction results on one of the test slices for all approaches along with ground truth mask.

### Lesion segmentation

Table 5 presents a summary of the evaluation metrics, including average Dice coefficient, IoU, precision, and recall scores computed on the test dataset. As indicated, the highest overall performance was achieved by BLS with BRS (OV), which attained the best scores across all metrics: a Dice coefficient of 0.640, IoU of 0.539, precision of 0.705, and recall of 0.640. Conversely, BLS without BRS exhibited the lowest performance, with scores of 0.414 for Dice, 0.328 for IoU, 0.586 for precision, and 0.354 for recall. The BLS with BRS (SLS) approach produced intermediate results, ranking second in performance with scores of 0.542 (Dice), 0.447 (IoU), 0.542 (precision), and 0.605 (recall).

**Table 5:** Evaluation metrics for different approaches

Methods	**Dice**$_{\text{avg}}$	**IoU**$_{\text{avg}}$	**Precision**$_{\text{avg}}$	**Recall**$_{\text{avg}}$
BLS without BRS (WV)	0.414	0.328	0.586	0.354
BLS with BRS (WV)	0.423	0.336	0.461	0.482
BLS with BRS (SLS)	0.542	0.447	0.542	0.605
BLS with BRS (OV)	**0.640**	**0.539**	**0.705**	**0.640**

To evaluate the number of FP and FN at a threshold of 0.5 on the test dataset, a comprehensive analysis was performed. Table 6 reports the counts of FP and FN volumes stratified by lesion size for each method. Notably, BLS with BRS (OV) demonstrated superior performance, yielding the lowest number of misclassified lesions larger than 20 mm$^3$, with only 35 FPs, and the fewest undetected lesions, with a single FN. In contrast, BLS without BRS and BLS with BRS (WV) showed comparable performance to each other but exhibited a higher incidence of FPs for lesions smaller than 10 mm$^3$ (totaling 20), and underperformed in detecting lesions larger than 20 mm$^3$, with five FNs. The remaining methods achieved intermediate results, but overall exhibited poorer detection performance. Additionally, the largest FPs (greater than 20 mm$^3$) for all approaches were predominantly observed in the early and late image slices, where imaging artifacts were present and the breast region was incompletely developed.

**Table 6:** Number of FP and FN lesion volume (mm^3^) for threshold = 0.5

Method	FPs			FNs		
	$\textrm{V} < 10$	$10 < \textrm{V} < 20$	$\textrm{V} > 20$	$\textrm{V} < 10$	$10 < \textrm{V} < 20$	V > 20
BLS without BRS (WV)	20	5	35	0	2	5
BLS with BRS (WV)	39	12	45	0	1	0
BLS with BRS (SLS)	25	11	46	1	1	2
BLS with BRS (OV)	11	9	35	1	2	1

**Fig. 11:**
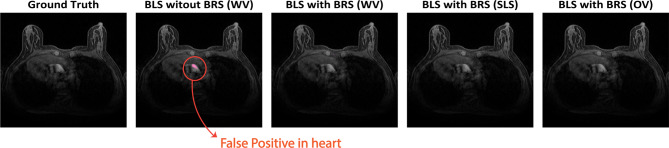
Illustrative examples of FP on the test dataset across different methods

Figure 11 illustrates an example of a FP occurring in a high-intensity region for BLS without BRS. The erroneously predicted lesion pixels are highlighted within a circle. In this instance, the heart was mistakenly classified as a lesion, underscoring the critical need to exclude noisy and high-intensity regions to minimize misclassification errors.

### Environmental impact

The CFP has become an increasingly critical concern over recent decades, and its relevance is particularly pronounced in AI applications [43]. Training large DL models demands substantial electrical power, which consequently results in significant carbon emissions associated with energy production [45, 46]. Therefore, addressing CFP considerations is an essential aspect of DL model development. Table 7 summarizes the TT for each approach, demonstrating that BLS with BRS (OV) achieves the shortest training duration, requiring only $16 \pm 4$ minutes per fold and fewer epochs to reach optimal performance. In contrast, BLS without BRS and BLS with BRS (WV) exhibit the longest TT, averaging $75 \pm 21$ minutes per fold.

**Table 7:** TT, CFP and last epochs across different approaches

Approach	TT per fold (min)	CFP per fold	Last epochs
BLS without BRS (WV)	75 $\pm$ 21	0.59 $\pm$ 0.17	30 $\pm$ 9
BLS with BRS (WV)	68 $\pm$ 13	0.54 $\pm$ 0.10	27 $\pm$ 5
BLS with BRS (SLS)	25 $\pm$ 12	0.20 $\pm$ 0.10	29 $\pm$ 14
BLS with BRS (OV)	16 $\pm$ 4	0.13 $\pm$ 0.03	24 $\pm$ 7

## Discussion

This study evaluates four distinct approaches for BLS, including one without BRS and three employing BRS. The results demonstrate considerable variation in model performance across validation and test datasets, particularly regarding the influence of shape modifications during training.

As detailed in Table 3, the data shape is significantly affected by the slice optimization approach. The most pronounced changes occur in the last two approaches, where dimensional adjustments extend beyond individual 2D slices to encompass the entire volume. Specifically, the OV approach achieves a more balanced data representation, downsizing 2D slices by approximately 46%, which starkly contrasts with the other methods. This substantial transformation is visually evident in Fig. 3.

In terms of performance, Table 4 highlights a clear advantage for the OV approach. Not only does OV demonstrate lower training loss, but it also sustains superior validation performance. Conversely, the WV approaches show negligible impact from BRS, suggesting that the exclusion of noisy regions in the whole volume does not substantially affect model performance. One plausible explanation is that over 70% of slices in the WV approach lack annotated lesions, biasing the model toward negative samples. In contrast, the selective inclusion of slices in OV and SLS promotes a more balanced learning process, enhancing both efficiency and segmentation quality.

A detailed analysis of lesion distribution, presented in Fig. 8, further elucidates key differences among the approaches. Lesion distributions for WV (with and without BRS) closely resemble those of SLS, as negative slices contain no lesion pixels. However, breast region maps (Fig. 9) reveal distinct variations. The SLS approach exhibits lower breast region concentration, resulting in a reduced $H_{\text{max} \vert \text{mid}}$ compared to WV-based methods. In contrast, the OV approach shows the highest density of lesion pixels along the x-axis, reflected in a unique intensity histogram. Notably, lesion distribution along the y-axis remains consistent across all approaches, with higher lesion aggregation observed in the left breast.

A broader comparison of breast region maps in Fig. 9 indicates that approaches other than OV display greater SD, attributed to less concentrated breast region distributions. Histogram intensity along the x-axis at $y_{\text{mid}}$ confirms that WV approaches yield the highest $H_{\text{max} \vert \text{mid}}$ values, whereas OV demonstrates a more localized distribution. These findings suggest that OV provides a more stable and spatially concentrated dataset, thereby improving segmentation efficiency.

The efficacy of BRS is further supported by the metrics in Table 5, where BLS with BRS achieves the best results, particularly in the OV approach. This conclusion is confirmed by Table 6, which analyzes FP and FN. The most substantial FPs (greater than 20 mm$^3$) occurred predominantly in early and late slices, attributable to artifacts and incomplete breast region development.

From a computational efficiency standpoint, optimizing the volume in the OV approach resulted in a marked reduction in TT and CFP. Compared to WV-based methods, this optimization decreased energy consumption by approximately 450%, highlighting the excessive computational cost associated with processing non-informative regions. Furthermore, the OV approach required fewer epochs to converge, demonstrating that a well-structured dataset enhances learning efficiency.

Future research should investigate whether adjusting the SD parameter for new datasets can further enhance segmentation outcomes. The SD parameter can significantly impact not only computational cost but also clinical relevance; therefore, identifying a generalizable SD value constitutes a critical avenue for future investigation. Additionally, alternative preprocessing techniques warrant exploration, as this study employed only fundamental preprocessing methods for medical images. Although increasing data diversity through multi-center datasets may improve model generalizability, lesion annotation remains a labor-intensive process requiring expert input. Since UNet++ was the sole DL model utilized in this study, future work should explore alternative modeling approaches like transformer-based models that could further optimize the efficacy of the OV method. While this study is confined to 2D approaches, extending the analysis to 2.5D and 3D methodologies presents a promising direction for assessing their relative performance and efficiency within the OV framework.

## Conclusion

In this study, a 2D DL model, UNet++, was developed to evaluate the impact of BRS on the performance of BLS. The model was trained separately using four different data shapes, each corresponding to a distinct ROI, to assess their influence on training efficiency. Consistent hyperparameters were maintained across all approaches, with an adaptive learning rate applied during training. A 5-fold cross-validation strategy, combined with a hybrid loss function, was employed to optimize the model’s parameters.

The results indicated that incorporating BRS over the entire volume yielded a modest improvement in model performance. More notably, optimization process derived from BRS substantially enhanced training efficiency. Among the BRS-based methods, segmentation utilizing the WV approach resulted in the lowest performance, whereas the OV-based segmentation achieved the best outcomes. Furthermore, the OV approach significantly reduced TT and was associated with the lowest CFP.

In conclusion, BLS with BRS using the OV method not only achieved superior performance but also demonstrated the greatest environmental sustainability by minimizing the CFP. This approach provides an eco-friendly solution for training on larger datasets while maintaining high accuracy, underscoring the importance of sustainability in DL applications.

## Data Availability

The Stavanger dataset analyzed in this study contains sensitive patient information and therefore not publicly available. It will be available upon reasonable request by contacting Endre Grøvik through the institution. The code for data preprocessing, data analyzing, visualization, modeling and evaluation is available on GitHub. To access the code repository, please follow the link on https://github.com/SamNarimani-lab/Breast.git.

## Abbreviations

AI: Artificial Intelligence
BLS: Breast Lesion Segmentation
BRS: Breast Region Segmentation
CFP: Carbon Footprint
CNN: Convolutional neural networks
DCE-MRI: Dynamic Contrast-Enhanced Magnetic Resonance Imaging
DICOM: Digital Imaging and Communications in Medicine
DL: Deep Learning
FN: False Negative
FP: False Positive
FPC: First Post-contrast
ML: Machine Learning
MRI: Magnetic Resonance Imaging
NIFTI: Neuroimaging Informatics Technology Initiative
OV: Optimal Volume
PC: Pre-Contrast
RAS: Right-Anterior-Superior
ROI: Region of Interest
SD: Safe Distance
SLS: Selected Lesion-containing Slices
TP: True Positive
WV: Whole Volume
